# Lower limb sagittal gait kinematics can be predicted based on walking speed, gender, age and BMI

**DOI:** 10.1038/s41598-019-45397-4

**Published:** 2019-07-02

**Authors:** Florent Moissenet, Fabien Leboeuf, Stéphane Armand

**Affiliations:** 1 Centre National de Rééducation Fonctionnelle et de Réadaptation - Rehazenter, Laboratoire d’Analyse du Mouvement et de la Posture (LAMP), Luxembourg, Luxembourg; 2 College of Health and Society, The University of Salford, Salford, UK; 3 Willy Taillard Laboratory of Kinesiology, University Geneva Hospitals and Geneva University, Geneva, Switzerland

**Keywords:** Biomedical engineering, Computational models

## Abstract

Clinical gait analysis attempts to provide, in a pathological context, an objective record that quantifies the magnitude of deviations from normal gait. However, the identification of deviations is highly dependent with the characteristics of the normative database used. In particular, a mismatch between patient characteristics and an asymptomatic population database in terms of walking speed, demographic and anthropometric parameters may lead to misinterpretation during the clinical process. Rather than developing a new normative data repository that may require considerable of resources and time, this study aims to assess a method for predicting lower limb sagittal kinematics using multiple regression models based on walking speed, gender, age and BMI as predictors. With this approach, we were able to predict kinematics with an error within 1 standard deviation of the mean of the original waveforms recorded on fifty-four participants. Furthermore, the proposed approach allowed us to estimate the relative contribution to angular variations of each predictor, independently from the others. It appeared that a mismatch in walking speed, but also age, sex and BMI may lead to errors higher than 5° on lower limb sagittal kinematics and should thus be taken into account before any clinical interpretation.

## Introduction

Clinical gait analysis (CGA) is nowadays fully integrated in the clinical decision-making for patients with complex gait disorders^1^. CGA attempts to provide an objective record that quantifies the magnitude of deviations from normal gait^2^. On this basis, a set of pathology-related impairments having the most impact on gait is identified and can be used to target the treatment^3^.

However, the identification of deviations is highly dependent with the characteristics of the normative database used. A special attention is then required to discriminate the differences between pathological and asymptomatic populations that could confound deviations. In particular, the gait of pathological populations is often observed at their own self-selected walking speed and compared to normative data established at the spontaneous walking speed of an asymptomatic population^4^. Since the spontaneous walking speed of pathological populations (*e*.*g*. ranged between 0.18 and 1.03 m.s^−1^ for stroke^5^) is often slower than for an asymptomatic population (ranged between 1.04 and 1.60 m.s^−1^ ^6^), a walking speed mismatch appears. Because walking speed is known to affect kinematics, kinetics, spatiotemporal parameters and muscular activity^7^, the identification of gait deviations can then become challenging since both pathology and walking speed difference may contribute to them^8^. This can be illustrated with the knee flexion amplitude during gait. A reduced knee flexion during the swing phase is commonly called stiff-knee gait after a central nervous system lesion, with a spasticity of the rectus femoris muscle as primary recognised causal impairment^9,10^. However, several authors have also pointed out the impact of walking speed on this gait feature^7,8^. The individual contribution assessment of this impairment is challenging when walking speed is not controlled^8^. But walking speed is not the only variable that could be a source of a mismatch in comparison of a patient and an asymptomatic population. Demographic and anthropometric parameters may also affect CGA interpretation. Recently, Chehab *et al*.^11^ demonstrated the impact of walking speed, but also age, sex and body mass index (BMI) on 3D kinematics and kinetics of the lower limb during gait. While walking speed was the most influential variable, the authors highlighted the influence of demographic and anthropometric parameters on very common parameters (*e*.*g*. pelvis tilt, peak of hip extension) used in the identification of gait deviations. In order to provide a breakthrough in identification of deviations in CGA and before considering a potential generalisation of automatic gait deviations detection algorithms^12^, a matching between the patient characteristics and an asymptomatic population database may be required.

However, the development of a new normative data repository requires a lot of resources and time. To overcome this issue, several methods have been proposed in the literature: 1) simple regression models between one or several independent variables (*e*.*g*. knee flexion angle) and one dependant variable (*e*.*g*. walking speed)^13–17^, 2) motion planning using parametric trajectories and a physics-based simulator^18,19^, 3) multiple regression models introducing several dependant variable (*e*.*g*. walking speed, demographic and anthropometric parameters)^20,21^. The first category is limited by the fact that only one dependant variable can be defined, while walking speed, demographic and anthropometric parameters may impact gait^11^. The second category offers a versatile framework to investigate the impact of many parameters on gait. However, to our knowledge, natural physiological pattern of walking have not yet been reproduced in such approaches^22^. The third category appears thus as an interesting approach to reproduce both the correlations between independent and dependant variables, and gait adjustments. This approach has already been used in an exploratory study by Koopman *et al*.^20^ on a limited number of participants (*i*.*e*. 15 healthy volunteers) and a reduced number of dependant variables (*i*.*e*. walking speed, body height) with encouraging results, and by Roislien *et al*.^21^ but only at normal and fast walking speeds (*i*.*e*. from 0.98 to 1.94 m.s^−1^).

This study relies on the assumption that normal gait can be predicted by a reduced number of parameters. The first aim of this study was to propose a method to predict lower limb sagittal kinematics with multiple regression models based on walking speed, gender, age and BMI as predictors. A robust multilinear regression was used to establish regressors (*i*.*e*. regression coefficients) and the results were evaluated using a leave-one-out cross validation. The second aim was to determine parameters that may have an influence on CGA interpretation (*i*.*e*. identification of gait deviations) in case of parameters mismatched with the normative database. This was achieved by applying the previously defined regressors for different values of one isolated predictor. The methodology applied in this study was illustrated on lower limb sagittal kinematics during gait for the sake of simplicity, but can be extended to all the parameters used in CGA (*e*.*g*. kinetics, EMG, spatiotemporal parameters).

## Results

### Determination of regression coefficients for each waveform

Details of each key-point used in this study can be found in Table 1. The regressors identified for each joint are reported in Table 2 (timing expressed as a percentage of gait cycle and angle) and as Supplementary information (angular velocity and acceleration).

**Table 1:** List of the key-points used to discretise kinematic waveforms. Columns ‘At/From’ and ‘To’ define the temporal phase during which each key-point is defined.

Joint	Notation	Item	At/From	To
Hip	HIS1	Angular value	Ipsilateral foot strike	
	HIS2	Angular value	Middle time between ipsilateral foot strike and ipsilateral foot off	
	HIS3	Minimum angular value	Ipsilateral foot strike	Ipsilateral foot off
	HIS4	Angular value	Ipsilateral foot off	
	HIS5	Maximum angular value	One quarter between ipsilateral foot off and next ipsilateral foot strike	Three quarters between ipsilateral foot off and next ipsilateral foot strike
	HIS6	Angular value	Next ipsilateral foot strike	
Knee	KNS1	Angular value	Ipsilateral foot strike	
	KNS2	Maximum angular value	Ipsilateral foot strike	Middle time between ipsilateral foot strike and ipsilateral foot off
	KNS3	Minimum angular value	Middle time between ipsilateral foot strike and ipsilateral foot off	Ipsilateral foot off
	KNS4	Angular value	Three quarters between ipsilateral foot strike and ipsilateral foot off	
	KNS5	Angular value	Ipsilateral foot off	
	KNS6	Maximum angular value	Ipsilateral foot off	Next ipsilateral foot strike
	KNS7	Angular value	Three quarters between ipsilateral foot off and next ipsilateral foot strike	
	KNS8	Angular value	Next ipsilateral foot strike	
Ankle	ANS1	Angular value	Ipsilateral foot strike	
	ANS2	Minimum angular value	Ipsilateral foot strike	Contralateral foot off
	ANS3	Angular value	Middle time between ipsilateral foot strike and ipsilateral foot off	
	ANS4	Maximum angular value	Ipsilateral foot strike	Ipsilateral foot off
	ANS5	Minimum angular value	Contralateral foot strike	Middle time between ipsilateral foot off and next ipsilateral foot strike
	ANS6	Maximum angular value	Ipsilateral foot off	Three quarters between ipsilateral foot off and next ipsilateral foot strike
	ANS7	Angular value	Next ipsilateral foot strike	

If a key-point is related to an instantaneous event (*e*.*g*. HIS1: the key-point is defined at the ipsilateral foot strike), only the related event is reported in column ‘At/From’. If a key-point is defined during a specific phase, the boundary events of the phase are reported in ‘At/From’ and ‘To’ columns, respectively (*e*.*g*. HIS3: the key-point is defined at the minimum angular value between the ispsilateral foot strike and the ipsilateral foot off).

**Table 2:** Regressors *β*_*i*_ defined for the timing and angle at hip, knee and ankle key-points (velocity and acceleration at the key-points are provided as Supplementary information).

Joint	Key-point	Parameter	${{\boldsymbol{\beta }}}_{{\boldsymbol{i}}}^{{\bf{0}}}$ *(Intercept term)*	${{\boldsymbol{\beta }}}_{{\boldsymbol{i}}}^{{\bf{1}}}$ *(Walking speed)*	${{\boldsymbol{\beta }}}_{{\boldsymbol{i}}}^{{\bf{2}}}$ *(Age)*	${{\boldsymbol{\beta }}}_{{\boldsymbol{i}}}^{{\bf{3}}}$ *(Sex)*	${{\boldsymbol{\beta }}}_{{\bf{i}}}^{{\bf{4}}}$ *(BMI)*	*RMSE*	*Predictors used*
Hip	HIS1	Timing (% gait cycle)	1	NS	NS	NS	NS	*0*	*(constant)*
		Angle (deg)	9.1713	20.4474	−0.0393	−4.8000	0.4698	*5*.*48*	*4*
	HIS2	Timing (% gait cycle)	37.0166	−10.9165	NS	−0.2067	NS	*1*.*28*	*2*
		Angle (deg)	−5.7088	7.0238	−0.0345	−4.7659	0.3042	*5*.*75*	*4*
	HIS3	Timing (% gait cycle)	58.4489	−14.7747	NS	0.7288	0.0829	*2*.*18*	*3*
		Angle (deg)	−11.9470	−17.0960	−0.0655	−6.4275	0.4643	*5*.*56*	*4*
	HIS4	Timing (% gait cycle)	73.4992	−21.8230	NS	−0.4234	NS	*2*.*49*	*2*
		Angle (deg)	2.7327	−29.6207	−0.0383	−5.9665	0.3500	*5*.*93*	*4*
	HIS5	Timing (% gait cycle)	90.0414	−3.1646	NS	1.0861	NS	*2*.*58*	*2*
		Angle (deg)	17.7667	13.8347	−0.0619	−5.4664	0.2803	*5*.*10*	*4*
	HIS6	Timing (% gait cycle)	101	NS	NS	NS	NS	*0*	*(constant)*
		Angle (deg)	9.1713	20.4474	−0.0393	−4.8000	0.4698	*5*.*48*	*4*
Knee	KNS1	Timing (% gait cycle)	1	NS	NS	NS	NS	*0*	*(constant)*
		Angle (deg)	−4.8743	1.4053	0.0702	−1.3015	NS	*4*.*22*	*3*
	KNS2	Timing (% gait cycle)	12.0826	6.6111	−0.0234	NS	NS	*2*.*36*	*2*
		Angle (deg)	−8.6072	31.3556	0.1093	NS	0.1168	*4*.*96*	*3*
	KNS3	Timing (% gait cycle)	34.9333	4.5361	0.0395	1.2407	NS	*3*.*74*	*3*
		Angle (deg)	−3.1995	−2.5919	0.0183	−1.2237	0.1868	*4*.*13*	*4*
	KNS4	Timing (% gait cycle)	56.0112	−16.3459	NS	−0.3331	NS	*1*.*89*	*2*
		Angle (deg)	5.0083	−5.4871	NS	−2.3842	0.1132	*3*.*91*	*3*
	KNS5	Timing (% gait cycle)	73.4992	−21.8230	NS	−0.4234	NS	*2*.*49*	*2*
		Angle (deg)	35.5374	−12.2774	0.0301	−2.5634	0.1980	*5*.*87*	*4*
	KNS6	Timing (% gait cycle)	77.0733	−7.4887	NS	−0.5444	NS	*2*.*03*	*2*
		Angle (deg)	41.6388	21.6639	NS	−1.7741	0.1663	*4*.*99*	*3*
	KNS7	Timing (% gait cycle)	94.2815	−5.5495	NS	−0.0796	NS	*0*.*69*	*2*
		Angle (deg)	3.4933	16.9053	NS	NS	−0.1651	*5*.*98*	*2*
	KNS8	Timing (% gait cycle)	101	NS	NS	NS	NS	*0*	*(constant)*
		Angle (deg)	−4.8743	1.4053	0.0702	−1.3015	NS	*4*.*22*	*3*
Ankle	ANS1	Timing (% gait cycle)	1	NS	NS	NS	NS	*0*	*(constant)*
		Angle (deg)	−1.2223	NS	NS	1.0414	NS	*2*.*96*	*1*
	ANS2	Timing (% gait cycle)	7.2097	NS	NS	NS	NS	*2*.*24*	*0*
		Angle (deg)	−4.6017	4.0205	NS	0.7755	−0.0965	*2*.*92*	*3*
	ANS3	Timing (% gait cycle)	37.0166	−10.9165	NS	−0.2067	NS	*1*.*28*	*2*
		Angle (deg)	7.0096	1.0447	−0.0086	NS	0.0789	*2*.*54*	*3*
	ANS4	Timing (% gait cycle)	51.8442	−14.3710	0.0580	NS	NS	*2*.*66*	*2*
		Angle (deg)	13.4480	−2.3965	0.0298	−0.4831	0.0765	*2*.*91*	*4*
	ANS5	Timing (% gait cycle)	72.0845	−15.6556	0.0197	−0.2129	NS	*1*.*99*	*3*
		Angle (deg)	−9.3038	−15.5580	NS	1.4280	NS	*6*.*31*	*2*
	ANS6	Timing (% gait cycle)	84.7634	NS	0.0160	0.7208	−0.0472	*3*.*40*	*3*
		Angle (deg)	7.4315	−2.8563	−0.0330	NS	NS	*2*.*91*	*2*
	ANS7	Timing (% gait cycle)	101	NS	NS	NS	NS	*0*	*(constant)*
		Angle (deg)	−1.2223	NS	NS	1.0414	NS	*2*.*96*	*1*

Only predictors having a statistical significant effect (p < 0.01) in the stepwise regression are used in the multilinear regression, and reported in this table (NS: not significant and not used in the multilinear regression). The root mean square error (RMSE) is given for each parameter as the average distance between original and predicted values using the defined multilinear regressions. Age and BMI are expressed respectively in years old and kg.m^−2^. Females are coded 0 and males coded as 1. Walking speed was expressed dimensionless by dividing the raw walking speed (m.s^−1^) by the square root of the product of the leg length (m) and the gravitational constant (m.s^−2^)^40^.

Each predictor was used at least in 48% of the regression equations (walking speed: 85%, age: 48%, sex: 70%, BMI: 50%), meaning that they were all of importance regarding kinematics. Except for one case (*i*.*e*. timing of ANS2), a regression equation was established using the selected predictors, and 2.7 predictors were used in average. The root mean square error (RMSE) of both the timing and the angle of each key-point were respectively 1.42 ± 1.19% of gait cycle and 5.55 ± 0.28° for the hip, 1.65 ± 1.32% of gait cycle and 4.79 ± 0.80° for the knee, 1.65 ± 1.30% of gait cycle, and 3.36 ± 1.31° for the ankle.

### Reconstruction of each waveform

**Figure 1:**
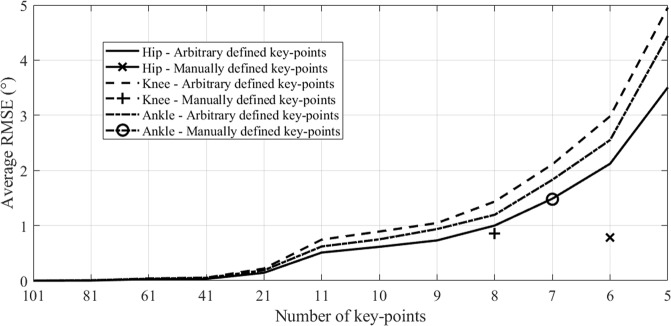
Impact of the number and type of key-points on the average root mean square error (RMSE) obtained across participants.

The RMSE related to the reconstruction error of the waveforms of hip, knee, and ankle joints were respectively 0.78 ± 0.48°, 0.86 ± 0.78°, and 1.48 ± 0.91°. These errors are highly correlated to the selection and number of key-points associated to each waveform (Fig. 1).

### Leave-one-out cross validation

**Figure 2:**
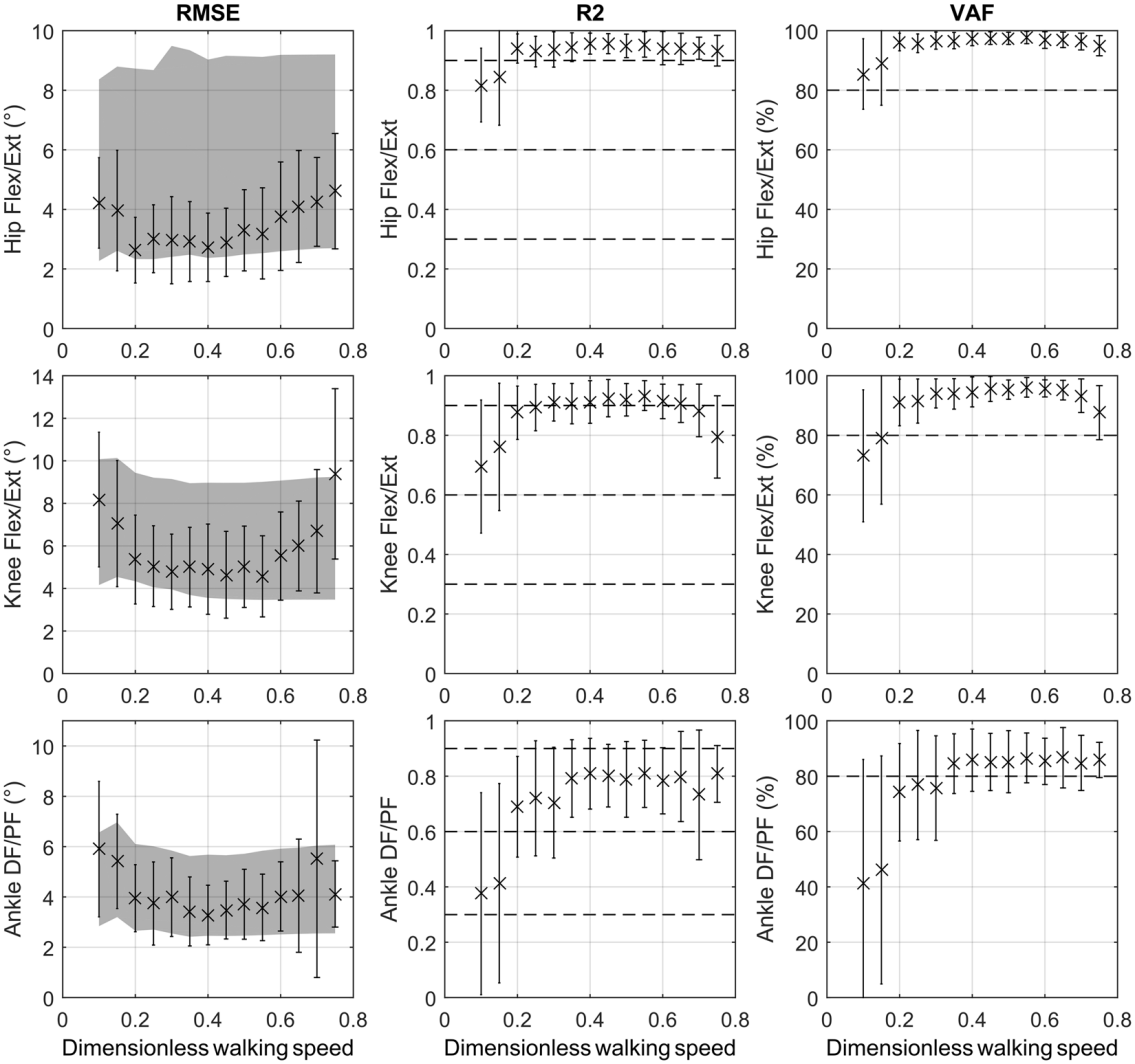
Results of the leave-one-out cross validation. From left to right: (1) Mean and standard deviation (SD) across participants of the root mean square error (RMSE) obtained for hip, knee, and ankle sagittal kinematics. The 1 SD corridor measured on the original waveforms is also reported (grey area) to show the variability observed across participants; (2) Mean and SD across participants of the coefficient of determination R2 obtained for hip, knee, and ankle sagittal kinematics. The [0;0.3], [0.3;0.6], and [0.6;0.9] ranges are shown (dotted lines) to highlight respectively poor, medium, and good correlation; 3) Mean and SD across participants of the Variance Account For (VAF) obtained for hip, knee, and ankle sagittal kinematics. The 80% of VAF level is reported (dotted line) for information.

The results of the leave-one-out cross validation are reported in Fig. 2.

On the whole, RMSE are included in the corridor of one standard deviation (SD) of the mean across all participants at different walking speeds. This corridor illustrates the variability contained in the original waveforms. This error is on average between 4° and 6°. However, a clear increase is observed for ankle and knee joints under 0.2 and beyond 0.7 dimensionless walking speed. These thresholds are also observed on the coefficient of determination (R2) and Variance Account For (VAF) results. Between these thresholds, correlations are good (between 0.6 and 0.9) to very good (beyond 0.9) and VAF is close or higher than 80%.

### Relative contribution of each predictor

**Figure 3:**
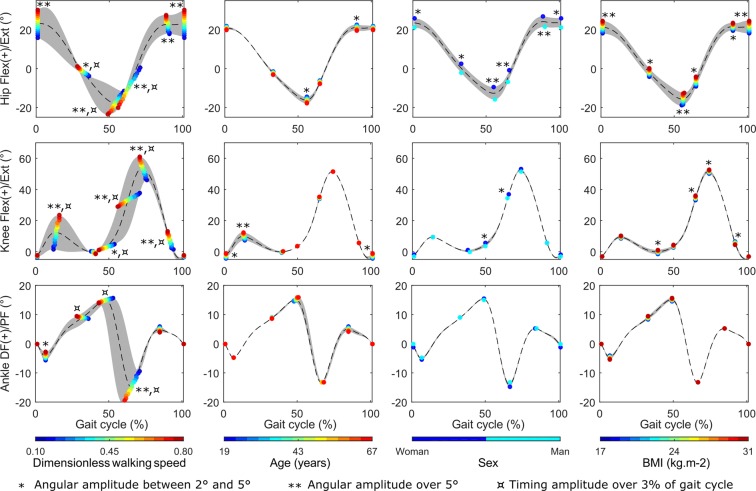
Relative contribution of each predictor to timing and angle of each key-point. Each column corresponds to the results obtained for the value variations of one predictor. Other predictors are set to their median value in our database. Grey areas and dotted lines corresponds respectively to the range and the median obtained for each waveform for each value variation of the predictor. The resulting key-points are reporting as a dot with a colour adjusted with the value of the selected predictor. Results are reported for hip flexion/extension (flex/ext), knee flexion/extension (flex/ext), and ankle dorsi/plantarflexion (DF/PF).

The relative contributions of each predictor on the key-points timing and angle are reported in Fig. 3. In particular, a difference in the results was considered as having a clinically significant impact if the maximal angular variation at a key-point was above 5°^23^, or if the temporal shift was above 3% of gait cycle^24^.

Concerning the hip, key-points HNS1, HNS3, and HNS5 correspond to the points proposed by Chehab *et al*.^11^. Timings were impacted by walking speed at HNS2-4 (reaching up to 15% of gait cycle). Angles were impacted by walking speed at HNS1 and HNS3-6 (reaching up to 20.7°), by sex at HNS3-5 (reaching up to 6.4°), and by BMI at HNS1, HNS3 and HNS6 (reaching up to 6.6°).

Concerning the knee, key-points KNS1-3 and KNS6 correspond to the points proposed by Chehab *et al*.^11^. Timings were impacted by walking speed at KNS2 and KNS4-7 (reaching up to 14% of gait cycle). Angles were impacted by walking speed at KNS2 and KNS5-7 (reaching up to 21.9°) and by age at KNS2 (5.3°).

Concerning the ankle, key-points ANS1-2 and ANS4-5 correspond to the points proposed by Chehab *et al*.^11^. Timings were impacted by walking speed at ANS3-5 (reaching up to 10% of gait cycle). Angles were impacted by walking speed at ANS5 (10.9°).

## Discussion

The objectives of this study were to propose a method to predict lower limb sagittal kinematics with multiple regression models based on a set of parameters (*i*.*e*. walking speed, gender, age and BMI) as predictors and to determine parameters that may influence the clinical interpretation in case of mismatch with the normative database. Indeed, it is of primary importance to distinguish the contribution of each perturbation that may alter the normal self-selected gait of an individual. These perturbations can be pathological, *i*.*e*. impairments, or not, *e*.*g*. walking speed. Not considering and removing the latter perturbations may lead to misinterpretation in the clinical practice^8,25^.

The use of a normative database matched in terms of walking speed, demographic and anthropometric parameters is a real challenge in CGA. Using a multilinear regression approach, our study proposed a method to avoid the need for a wide database including every variation and combination of these parameters. By applying this approach on a set of kinematic waveforms commonly used during the clinical interpretation of patients’ gait (*i*.*e*. hip, knee, ankle sagittal kinematics), we demonstrated that non-pathological gait parameters can be predicted from a reduced number of information (*i*.*e*. walking speed, age, sex, BMI) with an error within one standard deviation of the mean of the original data. More importantly, the regressors allowed us to assess the impact of the mismatch that commonly appears between a patient and a normative database in terms of walking speed, demographic and anthropometric parameters. To our knowledge, the present study is the first to quantify these errors that can dramatically impact the clinical evaluation. Thereby, we observed that walking speed may induce biases > 3% of gait cycle and > 5° on joint angles, *i*.*e*. thresholds recognised as critical for clinical interpretation^23,24^, when the normative database is not matched to the patient characteristics. Furthermore, even if the impact of age, sex, and BMI on gait features appeared less frequently than walking speed in our results, these predictors may also induce a bias > 5° on joint angles. A mismatch on one of these demographic and anthropometric parameters may thus be critical for clinical interpretation.

Prior to the multilinear regression approach suggested in this study, it must be noted that waveforms discretisation can be achieved with a reduced number of points while keeping a reasonable reconstruction error. As reported in Fig. 1, it is possible to define a set of key-points composed of 8 or less points to keep a reconstruction error below 2°, *i*.*e*. an error that may not have any consequence on data interpretation^23^. Indeed, in order not to obtain complex regression equations that would be less easily exploitable in clinical routine, it is preferable to define a low number of key-points. Of course, the reconstruction error tends to zero when the number of key-points meets the number of time frames. In this study, 6, 8, and 7 key-points were defined for the hip, knee, and ankle sagittal kinematics. This is similar to the method applied by Koopman *et al*.^20^ where 6 key-points were defined for each of these waveforms. It must also be noted that the manual selection of key-points can reduce the number of points needed to keep the error below the 2° threshold. Following the results of Chehab *et al*.^11^, we prioritised the use of local maxima and minima. These points often have a clinical meaning and appear thus as natural descriptors for the waveforms recorded during CGA. Moreover, these points are commonly interpreted as gait features^26^ and are used in the primary gait indexes^27–30^. The key-points selected in the present study demonstrated their importance when using the stepwise regression. For each point, almost 75% of the predictors had a statistically significant impact on the parameters (*i*.*e*. timing, angle, velocity and acceleration).

The multilinear regression approach used in this study was then evaluated using a leave-one-out cross validation. This validation method is particularly useful to estimate how accurately a model can predict data^31^. The error obtained across subjects and waveforms is similar to the one reported by Hanlon *et al*.^13^ with a simple regression approach with walking speed as predictor, and reported by Koopman *et al*.^20^ with a multiple regression approach with walking speed, squared walking speed, and body height as predictors. In particular, this error globally remains within 1 standard deviation (SD) of the mean of the original waveforms recorded on the participants. The amplitude of the 1-SD corridor in our original data is below 5° except for the hip sagittal kinematics. For this joint, the high variability might be related to the two primary sources of error in CGA: (1) Soft Tissue Artefacts (STA): corresponding to the motion of the skin, fat and muscles relative to the underlying bone, and (2) misplacement of some anatomical landmarks, which happens when the associated markers are not placed accurately on their anatomical locations^32–34^. The introduction of methods reducing or cancelling these errors might thus be interesting to improve the regressions based on these biased data^35,36^. More interestingly, our results highlight an increased error before and after the 0.2 and 0.7 dimensionless walking speed thresholds, respectively. In other words, the regression equations reported in our study are less able to reproduce the gait pattern measured at these ranges of speed. The 0.7 dimensionless walking speed threshold is already known as the preferred walk-run transition speed^37–39^ (the value of this threshold is 0.5 when the squared Froude number is used^38,40^). In term of motor control, this threshold represents a switch from a gait strategy to another one. However, Segers *et al*.^41^ demonstrated that this switch cannot be reduced to a sudden event but rather to a gradual process. These authors showed that a transition process exists and introduces discontinuities in kinematics. This could explain the increased error observed in our results over 0.7 dimensionless walking speed. The motor control adopted from this speed being no more related to the normal walking pattern, but to the transition phase between walking and running. It is also difficult to establish a good regression model with lower speeds. The literature about the 0.2 dimensionless walking speed threshold is sparse. However, Martin and Schmiedeler^39^ decided not to use dimensionless walking speed lower than 0.2 with their simple planar model since double support phase was too long at slower speeds. Indeed, at very slow speeds, walking becomes much more a succession of postures than a dynamic locomotion task. Postural control may thus be prioritised compared to the locomotion task. Smith and Lemaire^42^ also recently reported a discontinuity on several temporal parameters (*e*.*g*. stride, step, stance, and double support times) at 0.5 m.s^−1^, *i*.*e*. a dimensionless walking speed near 0.16. These authors suggested that this threshold could be related to a change in gait strategy, similarly to the one describing the walk-run transition. Regarding our present results, we can assume that between 0.2 and 0.7 dimensionless walking speed (*i*.*e*. 0.5 to 1.75 m.s^−1^), the prioritised locomotion strategy is walking. Beyond these thresholds, a transition may be induced by the central pattern generator to switch to a more adapted gait strategy, and an individual moving at a dimensionless walking speed below 0.2 may thus not “walk” anymore. While a linear regression model was able to accurately reproduce gait features between 0.2 to 0.7 dimensionless walking speed, a nonlinear approach may be required to describe transitions phases at low and high walking speeds. Further research is needed to verify this assumption. In particular, this limitation may currently restrict the clinical application of the proposed approach to patients with a highly reduced walking speed. Indeed, as reported in the introduction, the spontaneous walking speed of some neurologic patients can be below 0.2 dimensionless walking speed, e.g. after stroke^5^.

Using the established regressors, the proposed approach also allowed us to estimate the relative contribution to temporal shift and angular variations of each predictor, independently to the others. On the whole, most of the impacts of walking speed, age, sex and BMI observed in this study have already been reported in the literature. As already reported in the literature, we showed that walking speed impacts hip, knee, and ankle joints^7,11,15,20^. The temporal impact of walking speed has also already been described and it is known that a slower walking speed leads to a longer stance phase^7^, as observed in the present study. Furthermore, hip tends to be more flexed in women and when BMI increases, and the first peak knee flexion tends to be amplified with age. The effect of gender on lower limb kinematics has already been reported by several studies^11,43–46^. In particular, Cho *et al*.^44^ observed the same trend as in the present study for hip kinematics. Chehab *et al*.^11^ also reported an increased hip flexion in women, but only at the maximum flexion during swing (*i*.*e*. key-point HIS5). Cho *et al*.^44^ suggested that this amplified hip flexion could be related to a greater anterior pelvic tilt in women. This assumption is supported by the Wakayama Spine Study^47^ in which the pelvic tilt was shown statistically higher in women on lateral standing radiographs recorded on 1461 participants. The effect of BMI during gait has also already been investigated in the literature^11,48–50^. However, few studies reported a full description of lower limb kinematics, and if so, they did not necessarily matched walking speed between the different BMI groups^49^. To the best of our knowledge, only Chehab *et al*.^11^ reported the impact of BMI on lower limb kinematics on a rigorous basis. It results that BMI only has a significant effect on sagittal hip kinematics, with the same trend as in our study (*i*.*e*. the higher the BMI is, the more flexed the hip is). The impact of BMI on hip kinematics rather than distal joints could be partially due to the fact that the hip joint controls the motion and balance^51^ of body segments having the highest masses^52^ (*i*.*e*. pelvis/trunk and thigh). Finally, the observed increase of the first peak knee flexion with age is more difficult to explain. However, this increased flexion during stance was also reported by Chehab *et al*.^11^ and Roislien *et al*.^19^ in similar studies. Further studies might thus be necessary to explain this phenomena.

Our results must be interpreted carefully since this work has several limitations. Firstly, the number of participants is limited to 54 which remains an issue when defining subgroups (*e*.*g*. only 8 participants were aged between 50 and 60 years old). However, age, height, and weight distribution across participants was verified as following a normal distribution, ensuring a first level of quality in our regression equations. Furthermore, the range of each predictor was controlled so as to be representative of a healthy adult population. Secondly, a metronome was used to help participants to stay within predefined ranges of walking speed. While this rhythmic auditory stimulation may have been induced a specific cadence on participants, it has already been demonstrated that this stimulation may not impact lower limb sagittal kinematics^53^. Thirdly, the choice of the predictors was motivated by the current literature, in particular regarding the introduction of walking speed as predictor. However, it would have been interesting to differentiate cadence and step length (walking speed being the product of these variables) as recently proposed by Lim *et al*.^54^. Indeed, these authors reported that sagittal lower limb kinematics may primarily be impacted by a step length variation rather than a step frequency variation. This can be explained by the fact a change of step length induces a posture change, but not necessarily cadence. Similarly, BMI may be replaced by body height and weight, as well as body length could be split in leg length and upper body length. Other parameters such as emotions are also known to impact kinematics^55^, but such a consideration is above the scope of the present study. Fourthly, the number and choice of key-points remain manually defined. Regarding the whole process defined in the present study, it would be possible to optimise the number and choice of key-points to minimise the prediction error while keeping the number of key-points low for the sake of simplicity in terms of regression equations. Another method would be not to discretise kinematic waveforms using for example functional data analysis (FDA) as in the study of Roislien *et al*.^21^. However, we believe that the use of key-points with a clinical meaning may have a higher impact on the clinical interpretation and practice than a full waveform description that can be complex. Fifthly, the proposed approach has currently only be applied on sagittal lower limb kinematics. Future work could focus on extending the present study to other planes and other gait parameters, *i*.*e*. kinetics and muscular activity. Last but not least, the stepwise regression used in this study is known to have an overfitting issue. While a large dataset was used to establish our regression model and the leave-one-out cross-validation produced low RMSE and high R2 values, it would have been probably better to test the regression model on a dataset established on other participants to fully demonstrate that our model is not subject to overfitting. Further research should thus be considered to fully validate the proposed regression model.

To conclude, the multilinear regression equations proposed in this study can serve as a first basis to generate a reference gait profile matched to the patient walking speed demographic and anthropometric parameters. In case of diagnostic CGA, this reference would be a virtual normative database that can be obtained by varying the predictors based on the patients’ parameters. In case of longitudinal follow-up CGA, this reference would be a virtual subject similar to the current characteristic of the patient, without any pathology-related impairment. In both cases, the proposed approach could ease data and knowledge sharing in clinical practice and during multicentric studies by using a common normative database. Beyond these clinical applications, the present approach could be a promising approach to better understand gait maturation^56,57^ and the primary mechanisms of walking.

## Methods

**Figure 4:**
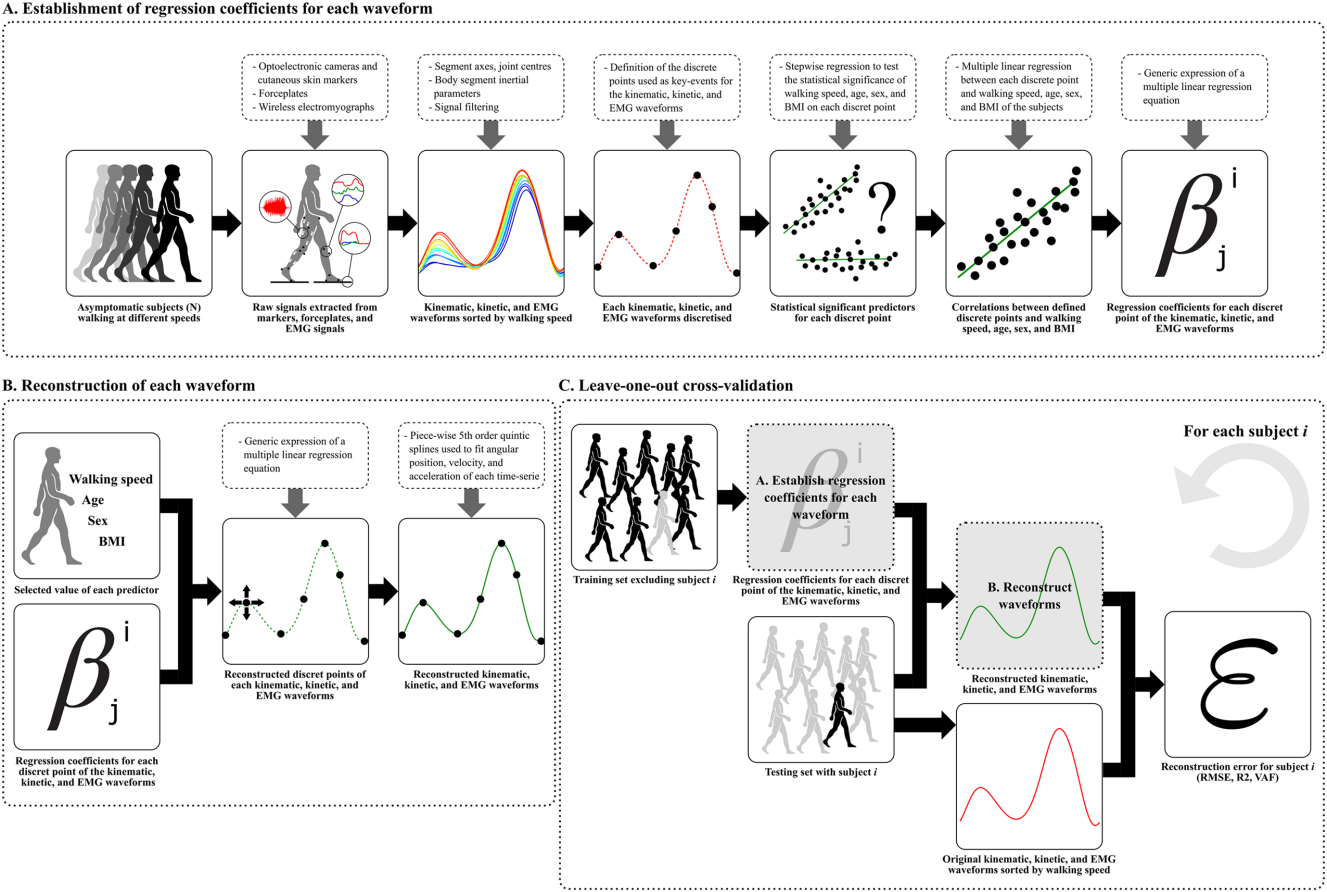
Flowchart of the multilinear regression process used in this study.

The whole process used in this study has been illustrated in Fig. 4.

### Reference database

Kinematic waveforms were collected as part of ongoing prospective studies in the Centre National de Rééducation Fonctionnelle et de Réadaptation – Rehazenter, Luxembourg^16,17,53,58^. Fifty-four adult participants (24 women –30 men) aged between 19 and 67 years (37.9 ± 13.7 years) and with a BMI between 17 and 31 kg.m^−2^ (height: 1.74 ± 0.10 m, 72.8 ± 13.3 kg) with no neuro-orthopaedic trouble were recruited. They all gave informed written consent prior to their inclusion and the protocol was conformed to the Declaration of Helsinki and approved by the Ethics Committee of the Rehazenter.

The 3D trajectories of 24 reflective cutaneous markers (*i*.*e*. bilateral iliac anterior and posterior spines, great trochanter, medial and lateral femoral epicondyles, peroneal head, tibial tuberosity, medial and lateral malleoli, 1^st^ and 5^th^ metatarsal, calcaneum) were recorded using a 10-camera optoelectronic system (OQUS-4, Qualisys AB, Sweden) sampled at 100 Hz. Markers were placed by anatomical palpation following the recommendation of Van Sint Jan^59^ by a unique expert operator. All data were post-processed with custom Matlab program (Matlab R2018a,The MathWorks, USA) based on the Biomechanical ToolKit (BTK)^60^. These trajectories were interpolated when necessary using a cubic spline and smoothed by a 4th-order lowpass Butterworth filter with a cutoff frequency of 6 Hz. The definition of joint centres and segment coordinate systems proposed by Dumas and Wojtusch^52^ were used and follow the recommendations of the International Society of Biomechanics (ISB)^61^. Joint kinematics was finally computed using the 3D Kinematics and Inverse Dynamics toolbox proposed by Dumas and freely available on the MathWorks File Exchange (https://nl.mathworks.com/matlabcentral/fileexchange/58021-3d-kinematics-and-inverse-dynamics).

The participants were asked to walk on a 10-m straight level walkway at five conditions of walking speed. During conditions C1, C2 and C3, the participants were asked to adapt their walking speed respectively between 0 and 0.4 m.s^−1^, 0.4 and 0.8 m.s^−1^, and 0.8 and 1.2 m.s^−1^. Several trials were allowed for training and a metronome was used to help participants to respect this speed constraint. It was assumed that this rhythmic auditory stimulation does not significantly impact kinematics as previously demonstrated by Schreiber *et al*.^53^. Conditions C4 and C5 corresponded to the participants’ spontaneous and maximal self-selected walking speed, respectively. One static and five gait trials (composed of one right gait cycle and one left gait cycle) were recorded for each participant and for each condition.

### Discretisation of kinematic waveforms

Only the sagittal kinematics of hip, knee and ankle joints were used in this study. The related waveforms were discretised according to the key-points defined by Chehab *et al*.^11^, and completed when necessary by technical points as proposed by Koopman *et al*.^20^. The complete list of the key-points used in this study are reported in Table 1. While a higher number of key-points would have produced reconstructed waveforms much close to the original ones (see Fig. 1), the choice was made to use key-points presenting a biomechanical and clinical meaning.

These key-points were selected to capture the curvature specificities of each waveform, *e*.*g*. minimal and maximal values, curvature inversion. The point at the beginning and the end of normalised gait cycle were used for every waveform. The angle and its first and second derivatives at the end of the gait cycle were set as equal to the values measured at the beginning of the gait cycle to ensure the continuity between cycles.

In parallel, the effect of the number and selection of the key-points on the quality of the waveforms reconstruction was assessed. Root mean square errors (RMSE) between the original waveform and the waveform reconstructed using the selected key-points interpolated by 5^th^ order piece-wise quantic splines^20^ were computed.

### Establishment of regression coefficients for each waveform

Regression coefficients – merged in the regressors vector ***B*** – allow to estimate a large set of parameters using a reduced number of predictors. In our case, walking speed, age, sex, and BMI, *i*.*e*. a set of demographic and anthropometric parameters, were used as predictors and merged in the predictors vector ***P***:

1 $${\boldsymbol{P}}=[{\boldsymbol{walking}}\,{\boldsymbol{speed}},\,{\boldsymbol{age}},{\boldsymbol{sex}},\,{\boldsymbol{BMI}}]{}^{T}$$

The impact of walking speed on gait has been highlighted in many studies in children^7^ and adults^8,62^. Kinematics, kinetics as well as muscle activity are known to be impacted by speed variations. In this sense, walking speed appears as an obvious predictor of gait features. Walking speed was computed by measuring the average velocity of pelvis markers in the walkway direction. It was expressed dimensionless by dividing the raw walking speed (m.s^−1^) by the square root of the product of the leg length (m) and the gravitational constant (m.s^−2^) (the result is called Froude number)^40^. Leg length was computed in standing position during static records as the distance between the iliac anterior marker and the medial malleolus marker of the same limb. Furthermore, Chehab *et al*.^11^ have recently identified on 121 asymptomatic subjects several gait features where variations were correlated with age, sex and BMI. These anthropometric and demographic parameters were thus also introduced as predictors in our study.

These predictors were used to estimate to estimate the timing ***t***_*i*_ expressed as a percentage of gait cycle, angle *θ*_*i*_, and its time derivatives ${\dot{\theta }}_{{i}}$ and ${\ddot{\theta }}_{{i}}$ of each key-point. These parameters were merged in the parameters vector ***X***. Derivatives ${\dot{\theta }}_{{\boldsymbol{i}}}$ and ${\ddot{\theta }}_{{i}}$ were introduced as proposed by Koopman *et al*.^20^ to reconstruct continuous waveforms. The regression model used in this study was thus expressed in the following vector form:

2 $${\boldsymbol{X}}={\boldsymbol{B}}\,{\boldsymbol{P}}$$

Before computing the regression coefficients related to ***X***, a stepwise regression (function *stepwisefit*, Matlab R2018a, The MathWorks, USA) was applied to test the predictors for each parameter of the key-point *i*. Only the predictors having a statistical significant effect (p < 0.01) were retained to estimate the regression coefficients. These predictors were merged in the significant predictors vector ${{\boldsymbol{P}}}^{\ast }$.

The regressors vector ${{\boldsymbol{B}}}_{{\boldsymbol{i}}}^{\ast }$ related to the significant predictors vector ${{\boldsymbol{P}}}^{\ast }$ was then obtained with a robust multilinear regression by using iteratively reweighted least squares with a bisquare weighting function (function *robustfit*, Matlab R2018a, The MathWorks, USA):

3 $$[\begin{array}{c}\vdots \\ \begin{array}{c}{{\boldsymbol{t}}}_{{\boldsymbol{i}}}\\ \begin{array}{c}{\theta }_{{\boldsymbol{i}}}\\ \begin{array}{c}{\dot{\theta }}_{{\boldsymbol{i}}}\\ {\ddot{\theta }}_{{\boldsymbol{i}}}\end{array}\end{array}\end{array}\\ \vdots \end{array}]=[\begin{array}{c}\vdots \\ \begin{array}{c}{}^{{\boldsymbol{t}}}{{\boldsymbol{B}}}_{{\boldsymbol{i}}}^{\ast }{{\boldsymbol{P}}}^{\ast }\\ {}^{\theta }{{\boldsymbol{B}}}_{{\boldsymbol{i}}}^{\ast }{{\boldsymbol{P}}}^{\ast }\\ \begin{array}{c}{}^{\dot{\theta }}{{\boldsymbol{B}}}_{{\boldsymbol{i}}}^{\ast }{{\boldsymbol{P}}}^{\ast }\\ {}^{\ddot{\theta }}{{\boldsymbol{B}}}_{{\boldsymbol{i}}}^{\ast }{{\boldsymbol{P}}}^{\ast }\end{array}\end{array}\\ \vdots \end{array}]$$

Finally, the root mean square error (RMSE) was computed for each parameter of each key-point as goodness-of-fit indicator of the regression, *i*.*e*. the average distance between original and predicted values from the defined multilinear regressions.

### Reconstruction of each waveform

Once the regressors are known, the parameters (*i*.*e*. timing *t*_*i*_, angle *θ*_*i*_, and time derivatives ${\dot{\theta }}_{{\boldsymbol{i}}}$ and ${\ddot{\theta }}_{{i}}$) of each key-point can be reconstructed for any value of the predictors. Kinematic waveforms were then obtained by a 5^th^ order piece-wise quantic splines interpolation, as proposed by Koopman *et al*.^20^ to avoid discontinuities in reconstructed waveforms.

The error related to the reconstruction process was evaluated. For that, original waveforms were discretised using the selected key-points, and the proposed reconstruction was applied. The RMSE between the original and reconstructed waveforms was then computed.

### Leave-one-out cross validation

The previously described methodology was validated using a leave-one-out cross validation. This was done using a custom Matlab program (Matlab R2018a, The MathWorks, USA). Such a validation allows us to estimate how accurate the regression equations established in this study will perform in a future use when applied on an independent dataset.

As descripted in Fig. 4, for each participant *j*, the validation process consisted in 1) establishing the regressors for each waveform based on a database composed of all participants excepted participant *j*, 2) reconstructing each waveform, 3) computing the RMSE (°), the coefficient of determination (R2), and the Variance Account For (VAF %) used as goodness of fit parameters to compared each reconstructed and original waveform of participant *j*.

### Relative contribution of each predictor

In order to assess the relative contribution of each predictor to the reconstructed waveforms, the following additional analysis was conducted. Each waveform was reconstructed by applying the previously defined regressors for different values of one selected predictor, whilst other predictors were set to their median value in our database. The range of values of the selected predictor was set from its minimum value to its maximum value in our database, with an interval allowing 15 conditions (except for the gender for which only 2 values can be defined).

The influence of each predictor on each key-point was then analysed in term of timing and angle. Rather than assessing a statistical significant difference between results, it was chosen to highlight any clinically significant difference. Concerning timing values, Bruening and Trager Ridge^24^ used a 4-frame window (*i*.*e*. 33 ms for data acquired at 120 Hz data) to compare several automated event detection algorithms. For an average gait cycle duration of 1.10 s in our database, this window corresponds to 3% of gait cycle. The maximum timing variation for the tested predictors’ values on each key point was thus considered as having a clinically significant impact above this threshold. Similarly, the maximum angular variation was classified following the angle ranges proposed by McGinley *et al*.^23^, *i*.*e*. below 2° no consequence is expected, between 2° and 5° consideration in data interpretation is needed, over 5° the results may mislead clinical interpretation.

## Supplementary information

Table S1

## Data Availability

All data and the custom Matlab program developed for this study have been made available on *zenodo*.*org* (10.5281/zenodo.1475166, https://zenodo.org/badge/latestdoi/141573910).
