# Supplementary material for: Lower limb sagittal gait kinematics can be predicted based on walking speed, gender, age and BMI
Source: Sci Rep. 2019 Jul 2;9:9510. doi: 10.1038/s41598-019-45397-4 (PMC6606631; doi:10.1038/s41598-019-45397-4)
Supplement: Supplementary file 1 — Table S1 [file 41598_2019_45397_MOESM1_ESM.docx]

**Title**: Lower limb sagittal gait kinematics can be predicted based on walking speed, gender, age and BMI.

**Authors**: Florent Moissenet^a, *^, Fabien Leboeuf^b^, Stéphane Armand^c^

**Authors affiliations**:

^a^ Centre National de Rééducation Fonctionnelle et de Réadaptation - Rehazenter, Laboratoire d’Analyse du Mouvement et de la Posture (LAMP), Luxembourg, Luxembourg

^b^ College of Health and Social Care, The University of Salford, United Kingdom

^c^ Willy Taillard Laboratory of Kinesiology, University Geneva Hospitals and Geneva University, Geneva, Switzerland

**Contact information**: Florent Moissenet, Centre National de Rééducation Fonctionnelle et de Réadaptation - Rehazenter, Laboratoire d’Analyse du Mouvement et de la Posture (LAMP), 1 rue André Vésale, 2674 Luxembourg, Luxembourg, +352 2698 94310, florent.moissenet@protonmail.com

**Supplementary information**

**Table S1:** Regression coefficients $\beta_{i}$defined for the velocity and acceleration at hip, knee and ankle key-points. Only predictors having a statistical significant effect (p < 0.01) in the stepwise regression are used in the multilinear regression, and reported in this table (NS: not significant and not used in the multilinear regression). The root mean square error (RMSE) is given for each parameter as the average distance between original and predictor values using the defined multilinear regressions. Age and BMI are expressed respectively in years and kg.m^-2^. Females are coded 0 and males coded as 1. Walking speed is expressed dimensionless by dividing the raw walking speed (m.s^-1^) by the square root of the product of the leg length (m) and the gravitational constant (m.s^-2^)^38^.

| **Joint** | **Key-point** | **Parameter** | $\beta_{i}^{0}$ *(Intercept term)* | $\beta_{i}^{1}$ *(Walking speed)* | $\beta_{i}^{2}$ *(Age)* | $\beta_{i}^{3}$ *(Sex)* | $\beta_{i}^{4}$ *(BMI)* | ***RMSE*** | ***Predictors used*** |
| --- | --- | --- | --- | --- | --- | --- | --- | --- | --- |
| Hip | HIS1 | Velocity (deg.s^-1^) | -0.6220 | 1.1067 | 0.0043 | NS | NS | *0.31* | *2* |
|  |  | Acceleration (deg.s^-2^) | 0.0690 | 0.0113 | NS | 0.0183 | -0.0049 | *0.07* | *3* |
|  | HIS2 | Velocity (deg.s^-1^) | -0.0171 | -2.1274 | -0.0015 | -0.0213 | NS | *0.19* | *3* |
|  |  | Acceleration (deg.s^-2^) | -0.0586 | 0.0455 | 0.0002 | NS | 0.0020 | *0.04* | *3* |
|  | HIS3 | Velocity (deg.s^-1^) | 0.0879 | NS | NS | 0.0199 | -0.0011 | *0.05* | *2* |
|  |  | Acceleration (deg.s^-2^) | 0.1675 | 0.0034 | NS | 0.0458 | -0.0013 | *0.05* | *3* |
|  | HIS4 | Velocity (deg.s^-1^) | 1.9518 | -0.8112 | NS | 0.0922 | NS | *0.29* | *2* |
|  |  | Acceleration (deg.s^-2^) | -0.1851 | 0.5060 | -0.0002 | -0.0276 | 0.0036 | *0.07* | *4* |
|  | HIS5 | Velocity (deg.s^-1^) | -0.0865 | 0.0707 | -0.0001 | 0.0096 | 0.0008 | *0.07* | *4* |
|  |  | Acceleration (deg.s^-2^) | -0.1675 | 0.0695 | NS | 0.0067 | 0.0023 | *0.06* | *3* |
|  | HIS6 | Velocity (deg.s^-1^) | -0.6220 | 1.1067 | 0.0043 | NS | NS | *0.31* | *2* |
|  |  | Acceleration (deg.s^-2^) | 0.0690 | 0.0113 | NS | 0.0183 | -0.0049 | *0.07* | *3* |
| Knee | KNS1 | Velocity (deg.s^-1^) | -0.7380 | 2.9304 | 0.0064 | NS | 0.0181 | *0.55* | *3* |
|  |  | Acceleration (deg.s^-2^) | 0.0398 | 0.4943 | NS | 0.0341 | -0.0046 | *0.17* | *3* |
|  | KNS2 | Velocity (deg.s^-1^) | -0.0365 | -0.0847 | -0.0003 | 0.0032 | NS | *0.07* | *3* |
|  |  | Acceleration (deg.s^-2^) | -0.0500 | -0.1991 | -0.0005 | 0.0065 | NS | *0.07* | *3* |
|  | KNS3 | Velocity (deg.s^-1^) | 0.0812 | 0.0046 | -0.0003 | -0.0122 | NS | *0.06* | *3* |
|  |  | Acceleration (deg.s^-2^) | -0.0019 | 0.2368 | 0.0002 | -0.0074 | NS | *0.05* | *3* |
|  | KNS4 | Velocity (deg.s^-1^) | 0.6119 | 0.9162 | NS | -0.0874 | -0.0067 | *0.26* | *3* |
|  |  | Acceleration (deg.s^-2^) | -0.0158 | 0.1028 | 0.0002 | -0.0180 | 0.0034 | *0.05* | *4* |
|  | KNS5 | Velocity (deg.s^-1^) | 2.8319 | 0.4654 | NS | 0.2078 | NS | *0.47* | *2* |
|  |  | Acceleration (deg.s^-2^) | -0.5936 | 1.0407 | NS | NS | NS | *0.16* | *1* |
|  | KNS6 | Velocity (deg.s^-1^) | -0.3570 | 0.3134 | NS | 0.0101 | NS | *0.16* | *2* |
|  |  | Acceleration (deg.s^-2^) | -0.5323 | 0.3984 | 0.0009 | 0.0299 | -0.0049 | *0.09* | *4* |
|  | KNS7 | Velocity (deg.s^-1^) | -1.8434 | -2.7403 | NS | NS | NS | *0.83* | *1* |
|  |  | Acceleration (deg.s^-2^) | 0.2682 | -0.2726 | 0.0018 | -0.0592 | 0.0085 | *0.17* | *4* |
|  | KNS8 | Velocity (deg.s^-1^) | -0.7380 | 2.9304 | 0.0064 | NS | 0.0181 | *0.55* | *3* |
|  |  | Acceleration (deg.s^-2^) | 0.0398 | 0.4943 | NS | 0.0341 | -0.0046 | *0.17* | *3* |
| Ankle | ANS1 | Velocity (deg.s^-1^) | -1.1670 | 0.7218 | 0.0025 | NS | NS | *0.36* | *2* |
|  |  | Acceleration (deg.s^-2^) | -0.1175 | 0.4622 | NS | -0.0251 | -0.0054 | *0.11* | *3* |
|  | ANS2 | Velocity (deg.s^-1^) | 0.1391 | -0.1507 | NS | NS | 0.0024 | *0.11* | *2* |
|  |  | Acceleration (deg.s^-2^) | 0.2049 | -0.1089 | NS | NS | 0.0042 | *0.09* | *2* |
|  | ANS3 | Velocity (deg.s^-1^) | 0.4599 | -0.3497 | -0.0015 | -0.0543 | 0.0073 | *0.17* | *4* |
|  |  | Acceleration (deg.s^-2^) | -0.0025 | 0.0283 | NS | -0.0048 | NS | *0.03* | *2* |
|  | ANS4 | Velocity (deg.s^-1^) | -0.0558 | NS | -0.0006 | 0.0064 | NS | *0.06* | *2* |
|  |  | Acceleration (deg.s^-2^) | -0.1325 | NS | -0.0016 | 0.0183 | NS | *0.09* | *2* |
|  | ANS5 | Velocity (deg.s^-1^) | 0.3972 | -0.3934 | NS | -0.0284 | 0.0042 | *0.24* | *3* |
|  |  | Acceleration (deg.s^-2^) | 0.8540 | -0.6255 | -0.0017 | -0.0446 | 0.0031 | *0.21* | *4* |
|  | ANS6 | Velocity (deg.s^-1^) | -0.1207 | 0.1484 | NS | 0.0099 | -0.0003 | *0.07* | *3* |
|  |  | Acceleration (deg.s^-2^) | -0.2313 | 0.2581 | NS | NS | NS | *0.08* | *1* |
|  | ANS7 | Velocity (deg.s^-1^) | -1.1670 | 0.7218 | 0.0025 | NS | NS | *0.36* | *2* |
|  |  | Acceleration (deg.s^-2^) | -0.1175 | 0.4622 | NS | -0.0251 | -0.0054 | *0.11* | *3* |
